# A comparison of the effects and usability of two exoskeletal robots with and without robotic actuation for upper extremity rehabilitation among patients with stroke: a single-blinded randomised controlled pilot study

**DOI:** 10.1186/s12984-020-00763-6

**Published:** 2020-10-19

**Authors:** Jin Ho Park, Gyulee Park, Ha Yeon Kim, Ji-Yeong Lee, Yeajin Ham, Donghwan Hwang, Suncheol Kwon, Joon-Ho Shin

**Affiliations:** 1 Department of Rehabilitation Medicine, National Rehabilitation Center, Ministry of Health and Welfare, 58, Samgaksan-ro, Gangbuk-gu, Seoul, Republic of Korea; 2 Translational Research Program for Rehabilitation Robots, National Rehabilitation Center, Ministry of Health and Welfare, Seoul, Republic of Korea

**Keywords:** Rehabilitation, Stroke, Upper extremity, Quality of life, Neurological rehabilitation, Stroke rehabilitation, Motivations, Exoskeleton devices, Robot, Robotic rehabilitation

## Abstract

**Background:**

Robotic rehabilitation of stroke survivors with upper extremity dysfunction may yield different outcomes depending on the robot type. Considering that excessive dependence on assistive force by robotic actuators may interfere with the patient’s active learning and participation, we hypothesised that the use of an active-assistive robot with robotic actuators does not lead to a more meaningful difference with respect to upper extremity rehabilitation than the use of a passive robot without robotic actuators. Accordingly, we aimed to evaluate the differences in the clinical and kinematic outcomes between active-assistive and passive robotic rehabilitation among stroke survivors.

**Methods:**

In this single-blinded randomised controlled pilot trial, we assigned 20 stroke survivors with upper extremity dysfunction (Medical Research Council scale score, 3 or 4) to the active-assistive robotic intervention (ACT) and passive robotic intervention (PSV) groups in a 1:1 ratio and administered 20 sessions of 30-min robotic intervention (5 days/week, 4 weeks). The primary (Wolf Motor Function Test [WMFT]-score and -time: measures activity), and secondary (Fugl-Meyer Assessment [FMA] and Stroke Impact Scale [SIS] scores: measure impairment and participation, respectively; kinematic outcomes) outcome measures were determined at baseline, after 2 and 4 weeks of the intervention, and 4 weeks after the end of the intervention. Furthermore, we evaluated the usability of the robots through interviews with patients, therapists, and physiatrists.

**Results:**

In both the groups, the WMFT-score and -time improved over the course of the intervention. Time had a significant effect on the WMFT-score and -time, FMA-UE, FMA-prox, and SIS-strength; group × time interaction had a significant effect on SIS-function and SIS-social participation (all, p < 0.05). The PSV group showed better improvement in participation and smoothness than the ACT group. In contrast, the ACT group exhibited better improvement in mean speed.

**Conclusions:**

There were no differences between the two groups regarding the impairment and activity domains. However, the PSV robots were more beneficial than ACT robots regarding participation and smoothness. Considering the high cost and complexity of ACT robots, PSV robots might be more suitable for rehabilitation in stroke survivors capable of voluntary movement.

*Trial registration* The trial was registered retrospectively on 14 March 2018 at ClinicalTrials.gov (NCT03465267).

## Introduction

Approximately 30–66% of stroke survivors suffer from upper extremity dysfunction, which leads to impediment of activities of daily living (ADL) and social participation [1]. Various interventions have been applied for upper extremity rehabilitation, and robotic rehabilitation has been recently popularised [2–4].

Robotic rehabilitation has potential advantages regarding the high repetition of specific tasks and interactivity, leading to active participation with less burden on therapists [2, 5]. Recent systematic reviews have suggested the beneficial effects of robotic rehabilitation on upper extremity dysfunction among patients with stroke [4, 6]. Veerbeek et al. described that robotic rehabilitation is more beneficial for the improvement of the motor control and strength of a paretic arm, but not for that of ADL, than is conventional therapy [6]. Furthermore, Mehrholz et al. demonstrated that robotic rehabilitation has more beneficial effects on ADL as well as on arm function and muscle strength compared to conventional therapy [4]. However, these conclusions should be considered cautiously because the robots that were included in these reviews were heterogenous: 28 and 24 different rehabilitation robots were included in the systemic reviews by Veerbeek et al. and Mehrholz et al., respectively. We recently showed that the use of end-effector and exoskeleton rehabilitation robots led to significant functional outcome differences stemming from distinct characteristics of the robots; this indicates that the differential effects might result from the inherent characteristics of the rehabilitation robot that was used [7]. In addition to the structural difference, the type of robotic control architecture (e.g., position, force, and impedance control) or robotic actuation (e.g., hydraulic power, pneumatic, and electric motor actuation) could also affect the therapeutic outcome [8, 9]. Nonetheless, there is a lack of studies that examined the differential effects according to the characteristics of robots. If the discrepant effects during upper extremity rehabilitation are understood according to the characteristics of robots, more suitable robotic rehabilitation may be applied and provided to each patient.

Accordingly, robotic devices can be classified as active-assistive and passive robotic devices according to the training modality. A passive robot does not provide assistive force, while an active-assistive robot provides assistive force with robotic actuators when the user is unable to make active movements [10–12]. Robotic active assistance is thought to be beneficial for users without voluntary movement because they can be trained with according to an ideal path or speed. Nonetheless, active assistance using manipulation for upper limb rehabilitation is too complex to be adopted with ease because the upper extremities are composed of several joints and different muscles, which allow movements with multiple degrees of freedom. Moreover, musculoskeletal problems associated with stroke such as spasticity, contractures, deformity, or hemiplegic shoulder pain make the application of robotic assistance more difficult. Additionally, excessive dependence on assistive force might interfere with active learning and participation for users who can perform voluntary movement. Therefore, we hypothesised that an active-assistive robot does not make a meaningful difference in terms of upper extremity rehabilitation relative to that made by a passive robot. Thus, we aimed to explore whether there is a difference in clinical and kinematic outcomes between active-assistive and passive robots during robot-assisted upper extremity rehabilitation of patients with stroke showing a Medical Research Council (MRC) scale score of 3 or 4 for the paretic proximal upper limb. In addition, we assessed the usability of robotic assistance. To our knowledge, this is the first clinical trial to directly compare rehabilitative effects between active-assistive and passive robots.

## Methods

### Study design

This was a single-blinded, randomised controlled pilot trial conducted at a single rehabilitation hospital. Participants were randomly assigned to the active-assistive robotic intervention (using an active-assistive exoskeletal robot with robotic actuators; ACT) group or passive robotic intervention (using a passive exoskeletal robot without robotic actuators; PSV) group in a 1:1 allocation ratio using a randomisation table calculated by the NCSS-PASS program. A researcher computed the randomisation sequence using the program, another researcher enrolled participants, and one other researcher assigned participants to interventions. Random allocation was conducted by using consecutive sealed opaque envelopes indicating group allocation, which were placed in a plastic container in numerical order. Each group completed 20 sessions of 30-min robotic intervention, 5 days a week for 4 weeks, conducted by an experienced research physical therapist in a research intervention room. Additionally, both groups received 30 min of conventional therapy for the affected upper limb, 5 days a week for 4 weeks. The study was approved by the institutional review boards of the hospital, and all participants provided written informed consent before enrolment. Our study was registered retrospectively with ClinicalTrials.gov (NCT03465267).

### Participants

This pilot study enrolled 20 patients with upper extremity dysfunction due to a stroke who were admitted in the rehabilitation hospital between March 2017 and December 2017. The inclusion criteria were: (1) age > 19 years; (2) the presence of hemiplegia owing to ischemic or haemorrhagic stroke; (3) stroke duration > 3 months; (4) hemiplegic shoulder and elbow flexion/extension with a Medical Research Council scale score of 3 or 4 for muscle strength; (5) the affected upper extremity Fugl-Meyer Assessment score (FMA) of 21–50; (6) shoulder and elbow flexor spasticity with the Modified Ashworth Scale score ≤ 1 +; (7) cognitive function of the level that facilitates the understanding and obeying of instructions of this study; and (8) the absence of a limited range of motion of the shoulder and elbow joint, as determined by the neutral zero method. The exclusion criteria were as follows: (1) a history of surgical treatment of the affected upper extremity; (2) a musculoskeletal problem of the upper extremity such as fracture, contracture, and shoulder subluxation of more than two finger breadth; and (3) cybersickness, which is the occurrence of nausea or vomiting when viewing a screen.

### Intervention

#### Active-assistive robotic intervention group

**Fig. 1:**
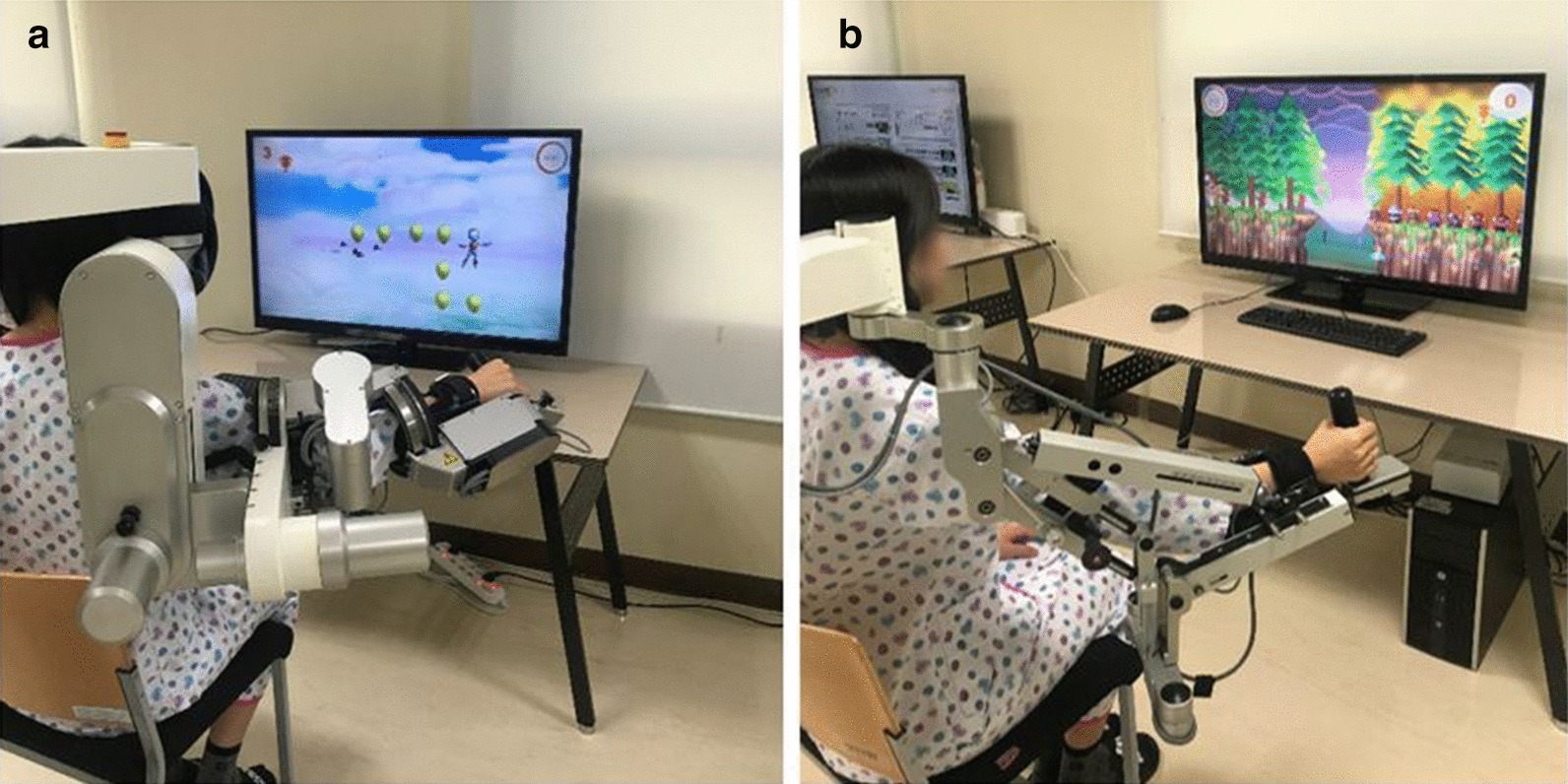
Two types of rehabilitation robots used for the robotic rehabilitation. **a** Armeo^®^ Power for the ACT group and **b** Armeo^®^ Spring for the PSV group. *ACT* active-assistive robotic intervention, *PSV* passive robotic intervention

In the ACT group, we administered the intervention using an Armeo^®^ Power (Hocoma Inc, Zurich, Switzerland) (Fig. 1a), which is a three-dimensional exoskeletal active-assistive robot used for upper extremity rehabilitation. Actuators actively assist the affected arm movement as an established extent, on top of arm weight support offsetting the device weight. Participants were trained with a game-based virtual reality environment with a focus on proximal upper limb movement.

#### Passive robotic intervention group

In the PSV group, we used an Armeo^®^ Spring robot (Hocoma Inc, Zurich, Switzerland) (Fig. 1b), which is an exoskeletal passive robot for three-dimensional upper extremity rehabilitation. The Armeo^®^ Spring provides gravity compensation, offsetting the device and the user’s upper extremity with the help of a spring but not with robotic actuators. Participants were trained under the same virtual reality environment as were those included in the ACT group.

### Outcome measure

We evaluated the FMA to measure impairment, Wolf Motor Function Test (WMFT) to measure activity, and Stroke Impact Scale (SIS) to measure participation according to the International Classification of Functioning, Disability, and Health (ICF) concept [13]. To determine more detailed kinematic outcomes, smoothness and mean speed were measured. Outcome measures were checked at baseline (T0), after 2 (T1) and 4 weeks of the intervention (T2), and 4 weeks after the end of the intervention (T3).

### Primary outcome

The primary outcome measure was the WMFT, which quantifies the upper extremity functional activity using 15 functional tasks [14]. The WMFT was chosen as the primary outcome measure because the purpose of this study was to investigate the improvement of the actual activity and participation with robotic rehabilitation. The WMFT is considered as an indicator of movement ability and activity [14, 15]. Additionally, the WMFT can also be used to measure subtle changes before and after intervention more sensitively and to avoid the ceiling effect considering the inclusion criteria of this study [14, 16]. The WMFT-score is rated on a 6-point scale, with the score ranging from 0 to 5; thus, the total score ranges from 0–75. The WMFT-time is the sum of the time required to perform all 15 tasks. A higher WMFT-score or shorter WMFT-time indicates better motor activity.

### Secondary outcomes

Secondary outcome measures were the FMA score, SIS score, and kinematic data. The FMA score, which ranges from 0 to 100, is a quantitative indicator of motor impairment following stroke, with higher scores reflecting a lower impairment [17]. We used the FMA-UE (shoulder, elbow, forearm, wrist, and hand; 33 items, 0–66) and FMA-prox (shoulder, elbow, and forearm; 18 items, 0–36). The SIS version 3.0, which is a stroke-specific self-reported questionnaire, has been applied as a health-related quality of life measurement tool to assess participation [18, 19]. We measured eight domains of SIS (strength, hand function, ADLs and instrumental ADLs [ADLs/IADLs], mobility, communication, emotion, memory and thinking, and social participation); the score of each domain ranges from 0 to 100; a higher score indicates a better health status. In the present study, four domains (strength, physical, ADLs/IADLs, and social participation) that are more relevant to proximal upper extremity function were selected for secondary outcome assessment. We also determined the SIS-overall (sum of scores of all eight domains) and SIS-function (sum of scores of ADLs/IADLs and social participation).

**Fig. 2:**
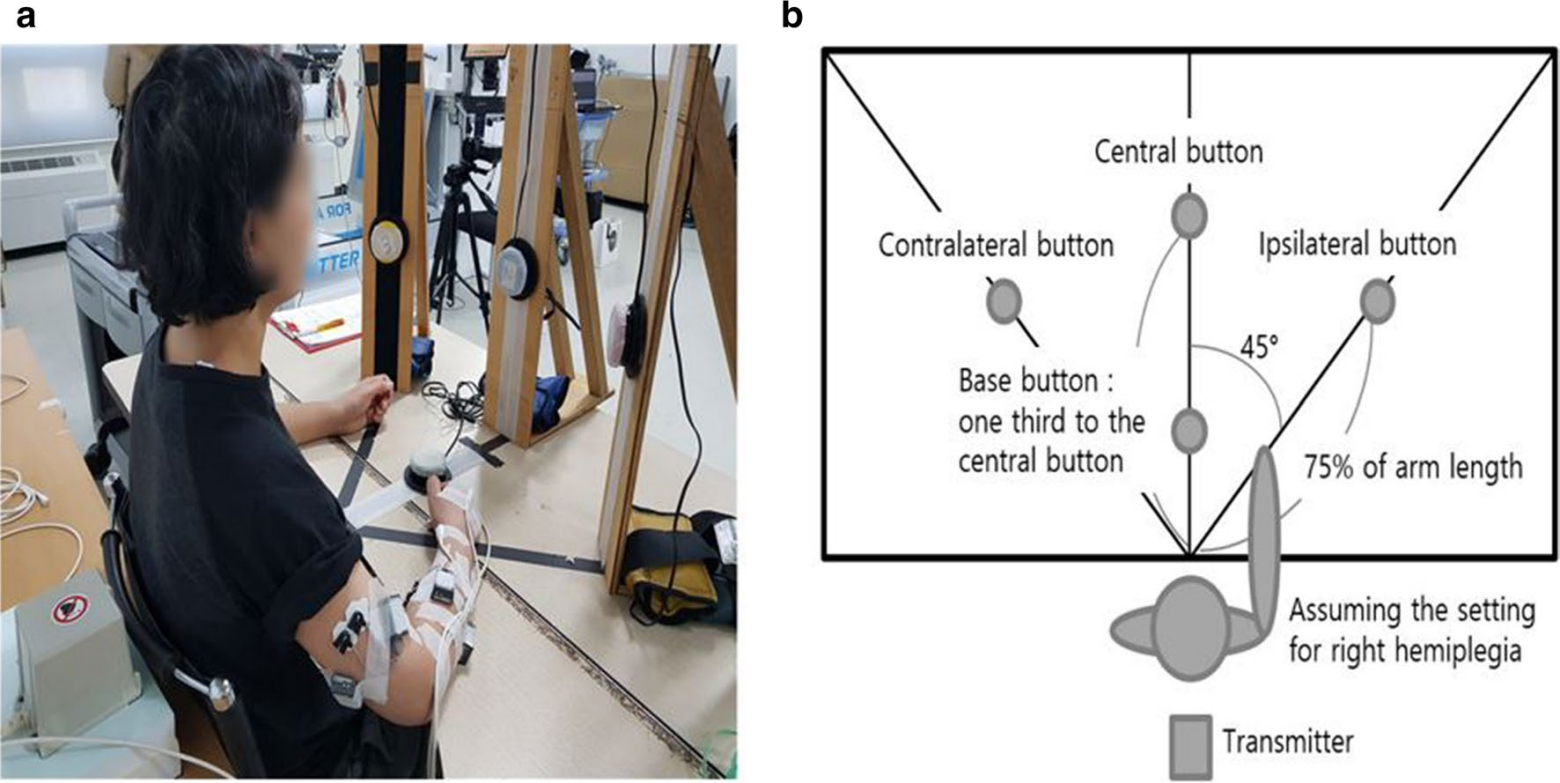
**a** A picture of the experimental setup for kinematic measurements. **b** Illustration of placement of the base button and target buttons

With regards to the kinematic analysis for detailed information on impairment, we recorded the position of the affected upper extremity using the trakSTAR™ system (Ascension Technology Corp, USA), which measures the movement of an electromagnetic sensor tracing 6 degrees of freedom (x, y, and z axes) at 80 Hz of sampling rate during each reaching movement. In the present study, the sensor was attached at the distal phalanx of the middle finger with double-sided tape, and the wire was fixed to the skin with bandage; the reference transmitter was located behind the participant (Fig. 2). Each patient was asked to sit in a chair in front of a table, the height of which was adjusted such that the elbow was flexed at an approximate angle of 90° in the sagittal plane; however, the distance of the table from the participant was maintained to ensure a comfortable reach. Participants practiced the reaching task three times to be familiarised with the experimental setup, which is described as follows. Buttons (base button and three target buttons) were positioned according to each participant’s affected arm length (from the distal end of the middle finger to the acromion). Three target buttons were set on a vertical wooden plate in front of the participant at the height of the participant’s xyphoid process and at a distance of 75% of the arm length in three different positions on the transverse plane (ipsilateral, central, and contralateral). The central button was installed in front of the midline, and two other buttons (ipsilateral and contralateral button) were placed in the ipsilateral and contralateral position at an angle of 45° from the central button. The base button was placed on the table in front of the midline at 25% of the measured arm length. Subsequently, participants were asked to reach from the base button to one of the three different target buttons, subsequently bringing back the upper limb to the base button at their own comfortable speed. Those movements were repeated nine times (three times to reach each target button in a randomised order) with 1 min of rest between each movement. Patients were instructed to limit trunk movements without a trunk restraint.

Subsequently, two kinematic performance indices were computed on the basis of the position data during reaching: spectral arc length (SAL) and mean speed (MSP). SAL is a dimensionless measure reflecting the smoothness, which was calculated using arc length of the Fourier magnitude spectrum of a movement speed profile [20]. A higher SAL value indicates a smoother and, thus, a better movement [21]. It is also known as an important marker reflecting motor recovery of patients with stroke [22]. The MSP was calculated by dividing the distance of the actual trajectory by the time required for reaching from the base button to each target button.

### Usability study

We assessed the usability of the patients with stroke based on individual interviews at the end of the intervention. Usability was also determined based on interviews conducted by the research physical therapists, who were in charge of the robotic intervention, and physiatrists, who observed the robotic rehabilitation at the end of the present study.

### Statistical analysis

We analysed the participants who completed outcome measurements at T2 at the least. When the results of T3 were not measured, the last observation carried forward method was used; thus, missed outcomes at T3 were filled in with those determined at T2. For the comparison of baseline characteristics between the two groups, Fisher’s exact test and the Mann–Whitney U test were applied for categorical variables and continuous variables, respectively. Repeated measures of analysis of variance (RM-ANOVA) were conducted using the group (ACT or PSV) and time (T0, T1, or T2) to compare the effect of each intervention across time, and time × group interactions were assessed. Greenhouse–Geisser corrections were applied when the violation of sphericity occurred. Additionally, the Mann–Whitney U test was performed for the intergroup comparison of kinematic data. A p-value of < 0.05 was considered statistically significant. All statistical analyses were performed using IBM SPSS Statistics for Windows, Version 20.0. Armonk, NY: IBM Corp.

## Results

**Fig. 3:**
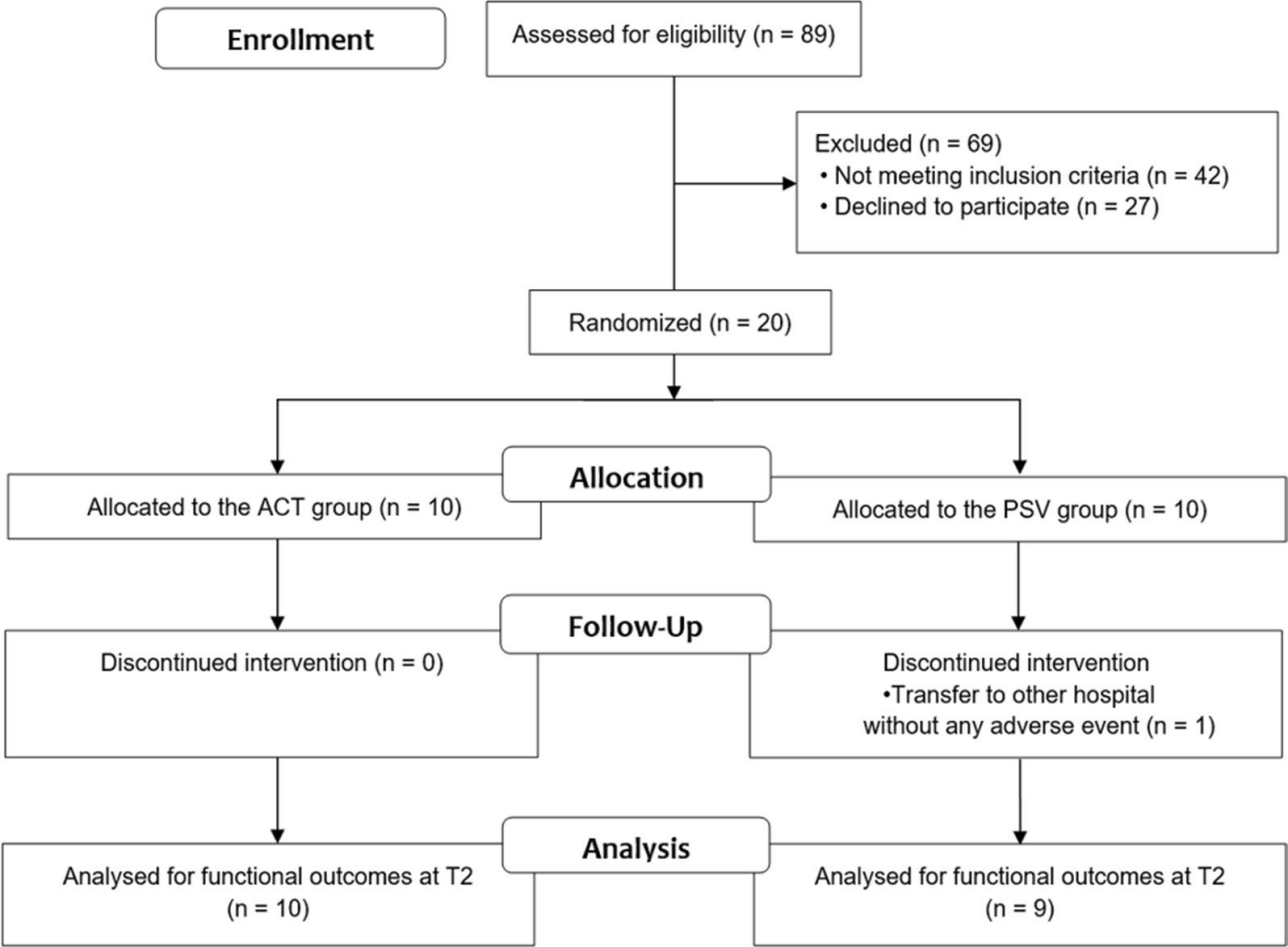
Flow chart showing the study design

A total of 20 patients with stroke participated in the present study from January 1, 2017, to December 31, 2017, and ten participants each were allocated to the ACT or PSV groups (Fig. 3). One participant of the PSV group dropped out because he was transferred to another hospital without any adverse events; thus, data on 19 participants (10 in the ACT group; 9 in the PSV group) who completed outcome measurements at T2 at the least were analysed (Table 1). The mean time after stroke onset were 11.8 ± 11.0 months in the ACT group and 9.6 ± 4.5 months in the PSV group. There was no statistical difference regarding the time after stroke onset between the two groups (p = 0.905).

**Table 1:** Baseline characteristics of the participants

	ACT group (n = 10)	PSV group (n = 9)	p-value
Age	54.9 ± 10.7	53.9 ± 16.7	0.842^a^
Time after stroke onset (month)	11.8 ± 11.0	9.6 ± 4.5	0.905^a^
Stroke type (infarction/haemorrhage)	5/5	4/5	1.000^*^
Hemiplegic side, right	6	5	1.000^*^
Sex, male	8	8	1.000^*^
FMA-prox	20.6 ± 5.0	22.2 ± 6.2	0.497^a^
FMA-UE	28.2 ± 10.9	30.2 ± 9.7	0.549^a^

Values are presented as the mean ± standard deviation or number

ACT, active-assistive robotic intervention; PSV, passive robotic intervention; FMA-prox, Fugl-Meyer Assessment-proximal (shoulder, elbow, and forearm; 18 items, 0–36); FMA-UE, Fugl-Meyer Assessment-upper extremity (shoulder, elbow, forearm, wrist, and hand; 33 items, 0–66)

^*^Fisher’s exact test

^a^Mann–Whitney U test

### Primary outcome

**Fig. 4:**
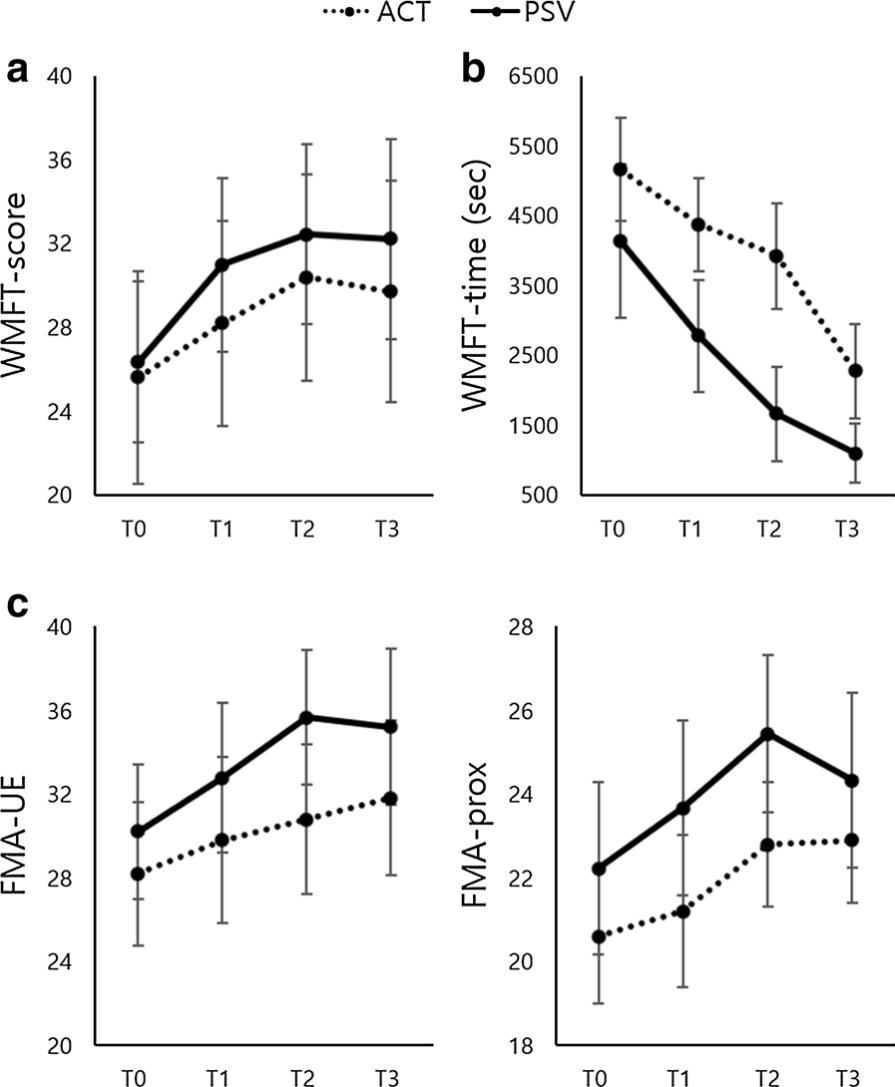
**a** WMFT-score, **b** WMFT-time, **c** FMA-UE, **d** FMA-prox. Values are presented as mean ± standard error. *ACT* active-assistive robotic intervention, *PSV* passive robotic intervention

Both groups showed similar tendencies: the WMFT-score improved over the course of the 4-week intervention and declined after its completion, whereas the WMFT-time continued to improve over time (Fig. 4). There was a significant effect of time on both the WMFT-score (F = 19.754, p < 0.001) and WMFT-time (F = 7.369, p = 0.002); however, there was no significant effect of group × time interaction on the WMFT-score (F = 0.700, p = 0.504) and WMFT-time (F = 0.802, p = 0.457).

### Secondary outcome

There was a significant effect of time on both the FMA-UE (F = 6.615, p = 0.004) and FMA-prox (F = 9.746, p < 0.001) without that of group × time interaction on the FMA-UE (F = 0.856, p = 0.434) and FMA-prox (F = 0.388, p = 0.682) (Fig. 4). Furthermore, group × time interaction had a significant effect on the SIS-function (F = 4.965, p = 0.013) and SIS-social participation (F = 6.388, p = 0.004), with more improvements in the PSV group than in the ACT group, but not on SIS scores (Table 2). Similarly, time had a significant effect on SIS-strength (F = 6.622, p = 0.004), but not on SIS-overall (F = 2.277, p = 0.118), SIS-function (F = 0.642, p = 0.532), SIS-physical (F = 1.909, p = 0.164), SIS-ADL/IADLs (F = 0.429, p = 0.655), and SIS-participation (F = 0.298, p = 0.744).

**Table 2:** Comparison of the performance between the ACT and PSV groups at T0, T1 and T2

Variable	ACT group (n = 10)			PSV group (n = 9)			Time * Group	
	T0	T1	T2	T0	T1	T2	F	p-value
SIS-overall	55.6 ± 12.2	57.9 ± 13.8	59.3 ± 14.1	59.0 ± 13.2	61.8 ± 14.3	63.7 ± 12.7	0.031	0.970
SIS-function	62.1 ± 16.7	56.0 ± 17.0	55.7 ± 15.6	59.5 ± 21.6	65.2 ± 20.8	71.5 ± 18.5	4.965	0.013
SIS-physical	37.3 ± 11.4	42.1 ± 12.6	44.9 ± 14.7	52.8 ± 14.8	49.3 ± 11.6	53.7 ± 16.0	1.765	0.187
SIS-strength	15.3 ± 13.7	23.0 ± 16.3	30.0 ± 18.6	32.1 ± 13.0	34.0 ± 20.9	44.4 ± 19.0	0.301	0.742
SIS-ADL/IADLs	62.6 ± 17.5	59.2 ± 18.8	63.4 ± 16.6	65.7 ± 20.3	67.2 ± 18.5	69.2 ± 24.0	0.261	0.772
SIS-social participation	61.5 ± 26.7	52.8 ± 30.3	47.9 ± 28.9	53.3 ± 24.8	63.1 ± 26.2	73.8 ± 24.4	6.388	0.004

ACT, active-assistive robotic intervention; PSV, passive robotic intervention; SIS, Stroke Impact Scale; IADLs, instrumental ADLs; ADLs, activities of daily living

**Fig. 5:**
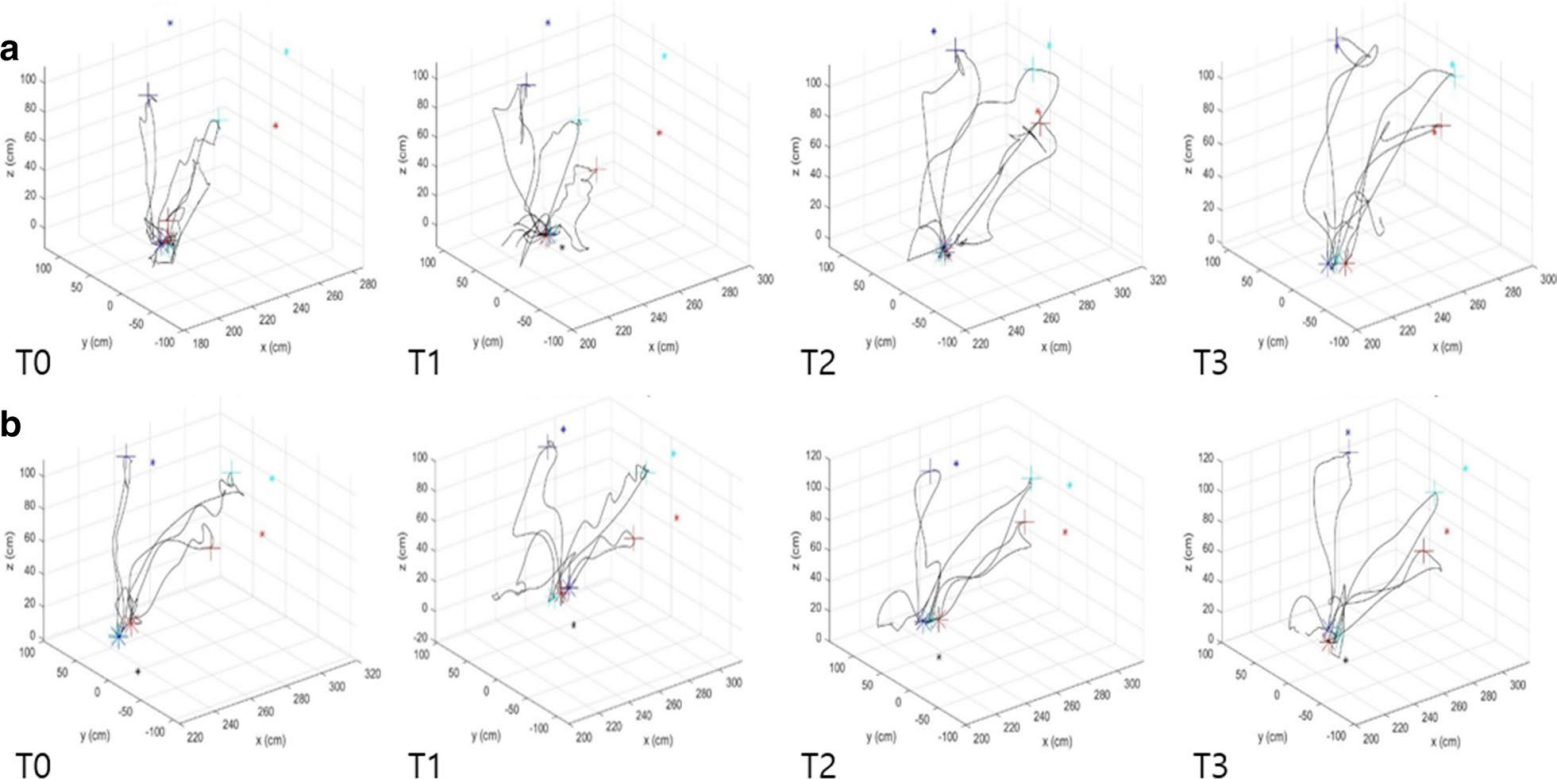
Examples of reaching trajectories across time from a patient with stroke in **a** the ACT group and in **b** the PSV group. *ACT* active-assistive robotic intervention, *PSV* passive robotic intervention

**Fig. 6:**
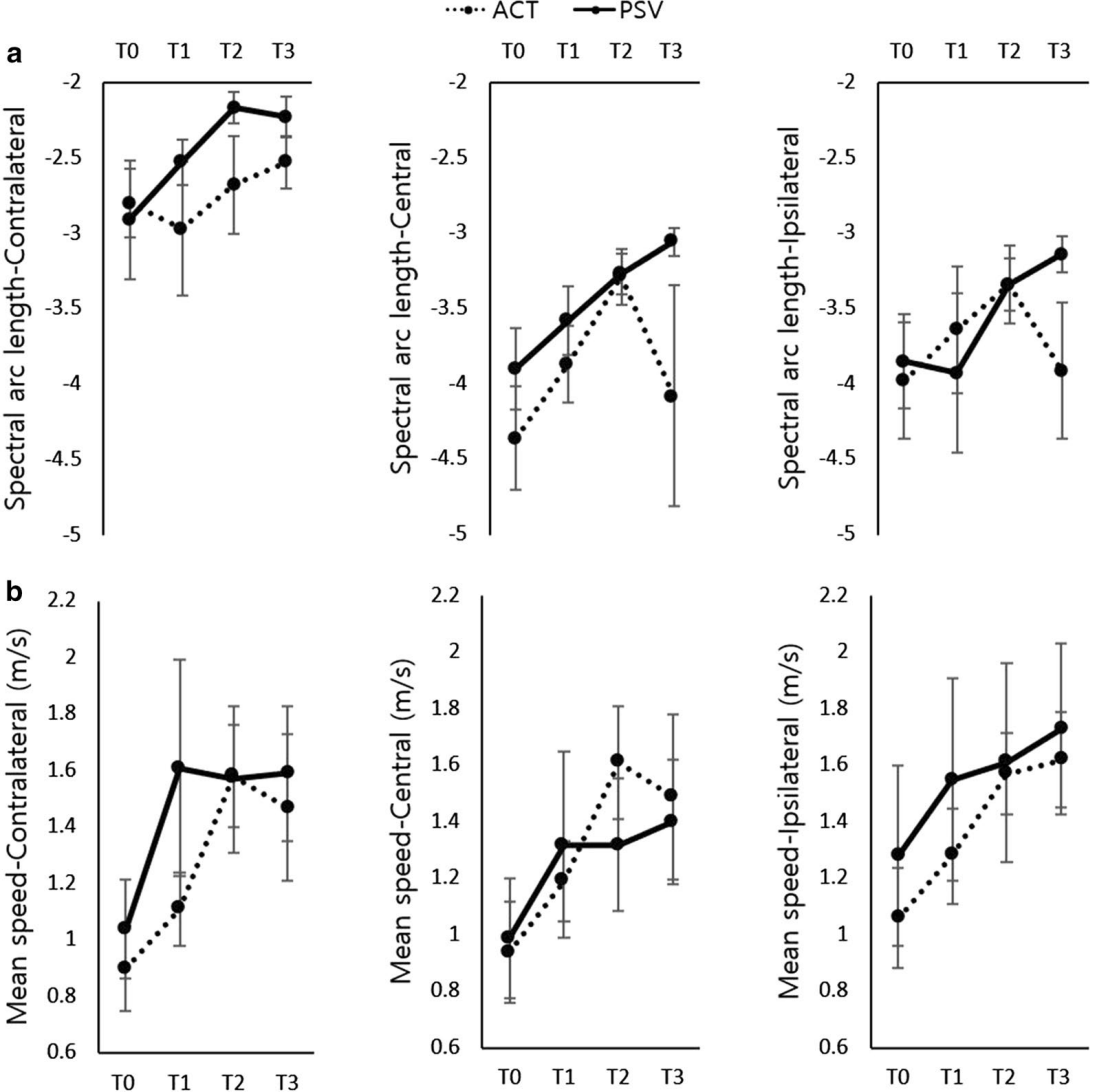
**a** Spectral arc length and **b** mean speed. Values are presented as mean ± standard error. *ACT* active-assistive robotic intervention, *PSV* passive robotic intervention

Kinematic data from 8 participants of the ACT group and 7 participants of the PSV group were available because of signal loss during the experiment (Figs. 5, 6) (Additional file 1: Table S1). Group × time interaction had no significant effect on SAL and the MSP across the target buttons, but time had significant effects on SAL-central (F = 9.589, p = 0.001), the MSP-contralateral (F = 12.707, p < 0.001), the MSP-central (F = 14.681, p < 0.001), and the MSP-ipsilateral (F = 7.323, p = 0.003). The PSV group showed better improvements compared to the ACT group with regard to SAL-ipsilateral from 2 to 8 weeks (p = 0.029) and from 4 to 8 weeks (p = 0.014), and with regard to SAL-central from 4 to 8 weeks (p = 0.029). On the contrary, the ACT group showed better progression of the MSP-central compared to the PSV group from 0 to 4 weeks (p = 0.021).

### Usability study

The usability, in terms of robotic active assistance, mechanical aspects of robot, the experience during robotic rehabilitation, and benefits of robotic rehabilitation, was summarised as pros and cons, separately for both groups in Table 3. Some patients felt that the robotic active assistance was beneficial for their training, as it afforded patient coordination and desirable movement pattern without aggravated compensatory movements of the trunk. However, active assistance was sometimes discordant to the patients’ intended movement. The mechanical complexity and high inertia stemming from the robotic actuators, which provide active-assistive force, make the robotic training more difficult. On the contrary, participants in the PSV group tried to invest more effort to move the limb compared to participants in the ACT group, which led to a sense of achievement, fulfilment, and motivation among participants because they could accomplish the tasks without external assistance.

**Table 3:** Usability test for each intervention from the patients with stroke, physiatrists, and therapists

ACT	PSV
Patients with stroke	
Pros	
Assistive force-as-needed function of the ACT robot facilitated the strengthening of the upper limb and increased smoothness of movement	The spontaneous and voluntary control of the robot seems to be linked to functional improvement in ADL The voluntary control of the robot without any external assistance leads to a feeling of achievement
Cons	
Assistive force sometimes gave the resistance for the intended voluntary movement The robotic exoskeleton was too heavy and bulky hampering arm movement	Assistive force-as-needed function might allow more optimal movement or the movement that was not possible without any assistance
Physiatrists and therapists	
Pros	
ACT robot seems to be better for introducing “ideal smooth and efficient” upper limb movement	More efforts were required from the participants; thus, self-motivated voluntary training was fulfilled
Cons	
Assistive force sometimes was not coordinated in terms of timing and context of the virtual environment The assistive force caused conflict with the spasticity of participants The inertia caused by manipulator was too high for the patients feeling heavier, paradoxically hampering upper limb movement	Compensatory movements were aggravated, such as abnormal posture or overuse of trunk instead of limb use, because of no assistance from the robots

ACT, active-assistive robotic intervention; PSV, passive robotic intervention; ADLs, activities of daily living

## Discussion

We demonstrated that the active-assistive and passive rehabilitation robots had distinct effects on different domains among patients with chronic stroke showing an MRC scale score of 3 or 4 for the affected proximal upper extremity muscle strength. In terms of the impairment and activity domains, there were no differences between the two groups. On the contrary, for the participation domain, the passive rehabilitation robot showed more beneficial effects compared to the active-assistive rehabilitation robot on the SIS-function and SIS-social participation. Kinematic analysis demonstrated that the PSV group showed better lasting effects on smoothness, while the ACT group showed immediate effects on speed.

Based on our findings, our results represented the effects of active-assistance during robotic rehabilitation because other factors of each group, such as the dose (time), task (three-dimensional task), and software platform (game-based virtual reality environment) were comparable. There have been few studies focusing on robotic rehabilitation using assistive force. A previous study compared active-assistive robotic reaching training and non-robotic free-reaching training [23]. In terms of clinical outcomes, no between-group differences were found. On the contrary, the kinematic analysis demonstrated that active-assistive robot training improves the smoothness but not the range of motion and straightness, indicating the subtle effects of active-assistive robot. A recent study compared the effects of robotic path assistance and/or weight support on upper extremity kinematics among patients with stroke [24]. They showed that path assistance led to a faster movement in the high functioning group and that a combination of path assistance and weight support led to a smaller error in the low functioning group. However, path assistance was not superior to weight support alone with regard to upper extremity kinematics of especially the lower functioning group, when considering a trade-off between speed and error.

Collectively, the results of previous studies and the current study indicate that active-assistive and passive rehabilitation robots showed no differences in their effects on clinical measures of parameters including impairment and activity, but they have distinct kinematic effects. There might be several explanations for these findings. First, our study population had the ability to move their affected arm without necessarily requiring external assistance, although some patients in the PSV group said that robotic active assistance might be more helpful for their training intensity and quality. Second, the active-assistive function was not sufficient to alleviate the fundamental issues of the upper limb function; therefore, other impairments or activities cannot be attributed to its effects [25]. The application of robotic assistance was not well coordinated with the motion of participants because of the inherent characteristics of the robot used in this study, thus impeding the intended voluntary movement in some patients, especially among those with spasticity. Thus, the therapists who participated in this study emphasised that the alignment of axis is important to minimise those conflicts. In order to avoid the discordance and convey more efficient assistance, various designs such as iterative learning impedance and safety motion decision making mechanisms have been introduced in the field of rehabilitation robotics [26, 27]. Therefore, the results of the present study should not be generalised to exoskeleton robots that were not used in this study. Third, a higher inertia owing to the weight of the manipulator that supplied assistance hampered the patient’s movement, thereby offsetting the effects of the assistance. In the usability study, participants in the ACT group complained that the device was ‘too heavy and bulky’, which seems natural considering that the ACT robot was an exoskeletal type with heavy robotic actuators [12]. Exoskeletal devices directly controlling each segment of the limb with robotic actuators tend to be large and bulky, and the inertia of the devices can only be resolved in part by themselves [28]. Fourth, the kinematic analysis had detected distinct features that were not explained by clinical scale scores [29].

Notably, the passive rehabilitation robot showed more beneficial effects with respect to the SIS-function and SIS-social participation compared to the active-assistive rehabilitation robot. Active assistance could induce ‘motor slacking’ of participants, which is a tendency to minimise metabolic and movement-related costs, thereby preventing active participation and simultaneously developing a dependence on the robot [30]. Motor slacking also possibly affects motivation, attention, effort, and active engagement, which are related to motor cortex excitability and motor plasticity [25, 31]. Robotic assistance in the ACT group decreased the loads on the participants’ motor systems, which impedes the learning of the fundamentals essential for performing the task [25]. On the contrary, the PSV group might experience more achievement, resulting in an improvement of participation, as reflected by the SIS, but not that of impairment and activity, as reflected by the FMA and WMFT. Similar results were found by a previous study that used a self-powered robot, which manipulated the participants’ affected arm using their unaffected arm and induced a higher degree of muscle activation in the affected arm than did externally powered robots, indicating the role of active participation [30]. In addition, those active engagements might induce learning and lasting effects, as shown by the lasting effects of smoothness after intervention in the PSV group.

Meanwhile, resistive training using robots have been applied in various studies, and the results have varied. Robotic upper limb resistive training was advantageous for the retention of motor learning compared to robotic-assistive training among healthy participants [32]. However, robotic upper limb resistive training for stroke survivors does not seem to be superior compared to the amount-matched robotic-assistive training in terms of motor function and strength [33]. Therefore, future studies on the effectiveness of robotic resistive training among stroke survivors are also needed.

There were several limitations to this study. First, for the PSV group, the passive robot also supports the limb with gravity compensation [34]. Nonetheless, most rehabilitation robots provide weight support of the limb to eliminate gravity effects; thus, our comparison represents the effects of active assistance during robotic rehabilitation and provides guidance for the development or application of active assistance rehabilitation robots. Second, the participants in this study may not be representative of all patients with stroke. The present study included patients with stroke showing an MRC scale score of 3 or 4 for the proximal upper limb strength, and therefore, the results may not be similarly applicable to other populations. For patients with limited muscle strength, active-assistive robotic training is necessary. In addition, this study enrolled only subacute and chronic patients by the inclusion criteria (stroke duration of > 3 months) in order to avoid the cofounding effects of spontaneous recovery [35–37]. It may be more difficult to observe significant differences between patients with stroke in the subacute or chronic phase in the two groups compared to patients in the acute phase with robust recovery [38]. In a systematic review regarding the electro-mechanical-assisted gait rehabilitation for patients with stroke, electromechanical-assisted gait rehabilitation may be helpful for patients in the acute phase but may not be helpful for patients in chronic phase [39]. Accordingly, our findings should be carefully interpreted because it might not be applicable for stroke survivors in the acute phase. Third, the number of participants may be insufficient to draw a definitive conclusion. In addition, a power analysis was not performed to calculate the required participant number because this was a pilot study. Fourth, the intervention dose was not sufficient to induce motor learning, as indicated by the decline of many outcomes after 4 weeks of treatment. According to a systematic review regarding robot-assisted arm training, the duration of intervention varied from 2 to 12 weeks [4]. Of the 19 studies included in the review, 8 studies adopted a 5- or 6-week intervention duration [4]. However, there are no definitive guidelines yet for the study period, and this study was conducted for only 4 weeks because of limitations of a pilot study and hospitalisation conditions of the participants. Our previous study comparing end-effector and exoskeleton rehabilitation robots for upper extremity rehabilitation also found significant differences with the same protocol; thus, 4 weeks of intervention duration was considered to be reasonable, although at least more than 20 h of extra repetitive task treatment could be beneficial in the previous study [40, 41]. Thus, further studies with a larger study population and a higher dose of intervention are needed.

## Conclusion

Our findings implied that active-assistive robots did not provide a significantly higher advantage compared to passive robots with regard to the improvement of impairment and activity. Active-assistive robots might have rather lower effects on participation, although there were differences with regard to kinematic results. Moreover, considering the complex nature and high price of active-assistive robots, passive robots could provide sufficient robotic rehabilitation for patients with stroke showing voluntary motor control of the upper limbs.

## Supplementary information

**Additional file 1: Table S1.** Comparison of the kinematic outcomes between the ACT and PSV groups at T0, T1, and T2.

## Data Availability

The dataset used in the present study is available from the corresponding author on reasonable request.

## Abbreviations

ADL: Activities of daily living
FMA: Fugl-Meyer Assessment
MRC: Medical Research Council
SAL: Spectral arc length
SIS: Stroke Impact Scale
WMFT: Wolf Motor Function Test
ACT: Active-assistive robotic intervention
PSV: Passive robotic intervention
