# Supplementary material for: A comparison of the effects and usability of two exoskeletal robots with and without robotic actuation for upper extremity rehabilitation among patients with stroke: a single-blinded randomised controlled pilot study
Source: J Neuroeng Rehabil. 2020 Oct 19;17:137. doi: 10.1186/s12984-020-00763-6 (PMC7574181; doi:10.1186/s12984-020-00763-6)
Supplement: Supplementary file 1 — Additional file 1: Table S1. Comparison of the kinematic outcomes between the ACT and PSV groups at T0, T1, and T2. [file 12984_2020_763_MOESM1_ESM.docx]

Additional file 1: **Table S1.** Comparison of the kinematic outcomes between the ACT and PSV groups at T0, T1, and T2

|  | ACT group (n = 8) | | | PSV group (n = 7) | | | | Time * Group | |
| --- | --- | --- | --- | --- | --- | --- | --- | --- | --- |
| Variable | T0 | T1 | T2 | | T0 | T1 | T2 | F | p-value |
| Spectral arc length-Contralateral | 2.8 ± 0.6 | 3.0 ± 1.2 | 2.7 ± 0.9 | | 2.9 ± 1.0 | 2.5 ± 0.4 | 2.2 ± 0.3 | 1.036 | 0.369 |
| Spectral arc length-Central | 4.4 ± 1.0 | 3.9 ± 0.7 | 3.3 ± 0.5 | | 3.9 ± 0.7 | 3.6 ± 0.6 | 3.3 ± 0.4 | 0.664 | 0.523 |
| Spectral arc length-Ipsilateral | 4.0 ± 1.1 | 3.6 ± 1.2 | 3.3 ± 0.7 | | 3.9 ± 0.8 | 3.9 ± 1.4 | 3.3 ± 0.5 | 0.236 | 0.792 |
| Mean speed-Contralateral | 0.9 ± 0.4 | 1.1 ± 0.4 | 1.6 ± 0.5 | | 1.0 ± 0.5 | 1.6 ± 1.0 | 1.6 ± 0.7 | 2.377 | 0.113 |
| Mean speed-Central | 0.9 ± 0.5 | 1.2 ± 0.4 | 1.6 ± 0.6 | | 1.0 ± 0.6 | 1.3 ± 0.9 | 1.3 ± 0.6 | 2.853 | 0.076 |
| Mean speed-Ipsilateral | 1.1 ± 0.5 | 1.3 ± 0.5 | 1.6 ± 0.4 | | 1.3 ± 0.8 | 1.6 ± 1.0 | 1.6 ± 0.9 | 0.599 | 0.557 |

ACT, active-assistive robotic intervention; PSV, passive robotic intervention.
